# Efficacy of Stem Cell Therapy in Amyotrophic Lateral Sclerosis: A Systematic Review and Meta-Analysis

**DOI:** 10.14740/jocmr2495w

**Published:** 2016-02-27

**Authors:** Mirian Conceicao Moura, Maria Rita Carvalho Garbi Novaes, Yuri S. S. P. Zago, Emanoel Junio Eduardo, Luiz Augusto Casulari

**Affiliations:** a Hospital Regional da Asa Norte, State Secretariat of Health of the Federal District, DF, Brazil; b School of Health Sciences, DF, and University of Brasilia, Brazil; c School of Medicine, School of Health Sciences, DF, Brazil; d University Hospital of Brasilia, DF, Brazil

**Keywords:** ALS, Amyotrophic lateral sclerosis, Motor neuron, Cell therapy, Survival

## Abstract

**Background:**

Published studies seeking to improve survival in amyotrophic lateral sclerosis (ALS) have poor results in humans, although there are several studies in animal models with positive results.

**Methods:**

We conducted a systematic review and meta-analysis of studies that were published between March 2009 and March 2015 on stem cell therapy and survival in animal models and patients with ALS. A total of 714 articles were identified, and from these, we selected preclinical *in vivo* studies and retrospective clinical studies.

**Results and conclusions:**

A meta-analysis confirmed the efficacy of stem cell therapy in improving survival in preclinical trials, where a mean difference of 9.79 days (95% confidence interval: 4.45 - 15.14) in lifespan favored stem cell therapy. In contrast, the number of clinical studies is still insufficient to assess their effectiveness, and these studies only demonstrate the absence of serious adverse events. However, even this conclusion should be interpreted with caution because clinical studies are retrospective and heterogeneous and have an unsatisfactory quality.

## Introduction

Amyotrophic lateral sclerosis (ALS) is the most common motor neuron disease. It is frequently sporadic and characterized by the progressive degeneration of both upper and lower motor neurons in the brain, brainstem and spinal cord [1]. The incidence of ALS varies between 1.2 and 4.0 per 100,000 individuals per year, and ALS predominantly occurs in males [2, 3]. Death occurs between 2 and 4 years after onset due to respiratory insufficiency [1].

The mechanisms of ALS development are poorly understood, but the injury mechanisms of the disease may include both glial cells and neurons [4, 5]. The main known mechanisms of ALS pathogenesis are oxidative stress with damage to RNA, mitochondrial dysfunction, impairment of axonal transport, glutamate excitotoxicity, protein aggregation, endoplasmic reticulum stress, abnormal RNA processing, neuroinflammation and excitability of peripheral axons [6].

The clinical heterogeneity of ALS makes it difficult to identify the exact cause of the disease for the development of effective therapies. However, the drug riluzole may extend patient survival by a few months [7]. In addition, multidisciplinary care, enteral nutrition and non-invasive ventilation can extend patient survival [8].

The results of studies on the use of stem cell therapy to preserve motor neuron function have been considered without any evidence of their effectiveness in humans, although the use of this therapeutic approach has been fairly efficacious in experimental animal models [9].

Given the need to identify new alternatives to treat ALS, the aim of the present study was to conduct a systematic literature review to assess the efficacy of stem cell therapy in clinical and preclinical studies.

## Materials and Methods

### Strategies used to identify studies for meta-analysis

In May 2015, we investigated primary preclinical *in vivo* studies and clinical studies and subsequent meta-analyses published between March 2009 and March 2015 in the following electronic databases: Medline, Embase, Cochrane Library and Lilacs. The following Medical Subject Headings (MeSH) and Health Science Descriptors (HScDe) were used: “Amyotrophic Lateral Sclerosis” OR “Motor Neuron Disease” AND “Treatment” AND “cell therapy”.

Two authors independently evaluated the titles and abstracts of all studies identified in the search of the aforementioned electronic databases based on the descriptors.

The inclusion criteria were the following: 1) clinical, prospective or retrospective studies of patients diagnosed with a motor neuron disease by means of anamnesis and electromyography according to the El Escorial [10] and Awaji criteria [11]; 2) preclinical and *in vivo* studies with assessment of survival compared to a control group, and studies of treatment after the onset of weakness; and 3) studies based on the use of stem cell therapy to increase survival time compared to placebo or other treatments used by the control group.

The exclusion criteria were studies in which participants presented with respiratory failure or spinal muscular atrophy, studies in which the treatment was administered only prior to the disease onset, and narrative reviews, letters, editorials, case reports, duplicate publications or those without objective data to be evaluated.

Articles published in all languages were included.

The studies that met the inclusion criteria were obtained in full. References were also considered, and communication with the authors was established in cases of doubt. Disagreements were resolved by consensus, and when this was impossible, there was a subsequent analysis by two additional reviewers.

### Data extraction

Data were obtained from each study using a review form with the following content: author, place where the work was conducted, year of publication, intervention, study design, number of participants, age, analysis by intention to treat, declaration of conflict of interest, evaluation by a research ethics committee, and animal species used if the study was preclinical. The following outcomes were assessed: 1) comparison between different treatments; 2) analysis of mean survival and absolute days of survival; 3) mean duration of the disease until the start of the intervention; 4) incidence of reactions and adverse effects of proposed treatments.

### Assessing the quality of the studies

Quality was assessed by two independent authors, and in cases of disagreement, the situation was resolved by consensus among all authors. The Grading of Recommendations, Assessment, Development and Evaluation (GRADE) model [12] was used to access the quality of the studies. The following data were observed in the studies: 1) methods including research question, treatment sequence, allocation confidentiality, post-intervention follow-up, blinded outcome assessment, primary clinical outcome measures, location of study, protection against contamination, calculation of statistical power, sample representativeness, conflict of interest, and ethical aspects; 2) participants including inclusion criteria, exclusion criteria, age, gender, disease severity, and disease variants; 3) interventions including procedures, follow-up time, and method for monitoring disease progression; and 4) outcomes assessed in the review including disease duration before intervention and survival time.

The results of the primary outcome were obtained based on the intention-to-treat principle. For a continuous outcome, the following variables were calculated: mean, standard deviation and number of participants in each group. Data from work that was published more than once were obtained from the more thorough study.

The authors rated each primary study according to the overall quality of evidence as follows: A - high; B - moderate; C - low; and D - very low, assigning scores of 1 - 5 according to the number of biases.

For analytical purposes, the studies were grouped as 1) interventions in animal models and 2) clinical studies.

### Statistical analysis

Statistical analysis was performed in preclinical trials using RevMan software, version 5.3. All P-values < 0.05 were considered to be statistically significant.

For continuous variables, such as animal survival in days, the weighted mean difference (random effects model) was calculated based on the DerSimonian and Laird method, with a corresponding 95% confidence interval (CI).

To evaluate the heterogeneity among the studies, a heterogeneity test was performed by calculating both the Q-test of heterogeneity and the I^2^ test of inconsistency. Heterogeneity was considered significant when P < 0.10. In addition, a sensitivity analysis was conducted using a funnel plot to quantify the presence of publication bias.

## Results

Initially, 714 articles were obtained. Based on their abstracts, we selected one retrospective controlled clinical trial, 14 preclinical studies and 12 clinical uncontrolled descriptive studies.

After each original document was reviewed and data were obtained, four preclinical trials were excluded. Of these, one was an *in vitro* study [13], two did not include a quantitative evaluation of survival [14, 15], and one only reported survival ratios and proportions [16].

**Figure 1:**
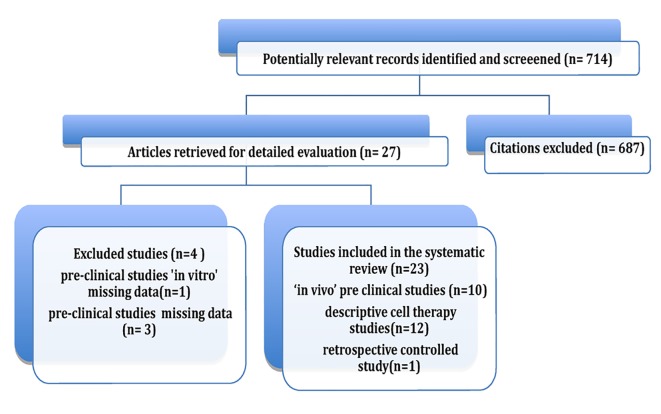
Review flowchart.

Finally, nine preclinical *in vivo* studies and 12 retrospective descriptive studies using stem cell therapy were included in the review. Although most of these studies were not controlled and randomized, they were included due to the limited number of studies that met this requirement. A flowchart illustrates the selection process that was adopted in the systematic review (Fig. 1).

### Preclinical studies

Preclinical studies have been insightful for identifying the types of stem cells that offer therapeutic benefit in ALS. *In vivo* studies were conducted in transgenic mice expressing human mutated superoxide dismutase 1 and G93A^(SOD1G93A)^ and presented experimental ALS treatments using neuroprotective therapies. These studies can be considered homogeneous with respect to their methods and their evaluation of outcomes in animals, which allows for a comparison using meta-analytical methods that involve a network meta-analysis [12]. All included studies analyzed survival using the Kaplan-Meier method with a log-rank test, but only those studies that clearly included the mean survival of animals in days and standard deviation were included in our meta-analysis. The authors of all of the studies that did not include such data were contacted via email.

**Figure 2:**
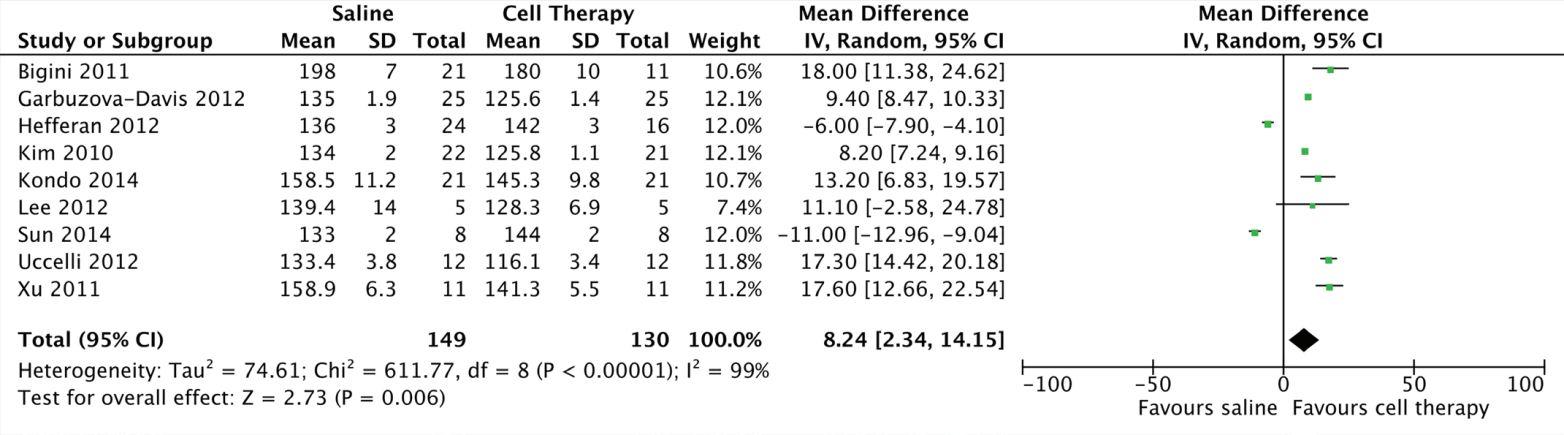
Preclinical studies in amyotrophic lateral sclerosis model with stem cell therapy, 2009 - 2015.

Figure 2 shows an acyclic graph (forest) of cell therapy preclinical trials in animal models, demonstrating a mean meta-analytical difference of survival of 9.79 days (95% CI: 4.45 - 15.14), which favored cell therapy over placebo.

In 2010, a study conducted in South Korea [17] showed that mesenchymal cell transplantation (ALS-hMSCs) at three different doses in association with cyclosporine was effective in slowing disease progression at high doses using an intrathecal injection of 1 × 10^6^ cells/mL into the cisterna magna (P = 0.033) and an intravenous injection of neurotrophic factors. In 2011, in Italy [18], intraventricular administration of mononuclear cells from human umbilical cord showed that treated animals had a significantly longer survival than controls (P < 0.01, Gehan-Breslow-Wilcoxon test). A study in 2012 [19] showed that an intravenous injection of mesenchymal cells also led to increased survival of the animals (P < 0.005). In 2014, a study in Japan showed the efficacy of a transplantation of glial-rich neural progenitors in attenuating motor neuron degeneration and disease progression in rodent models [20].

In 2012, Garbuzova-Davis et al [21] performed weekly intravenous injections of cells from human umbilical cord in the pre-symptomatic stage and in the 13th week of the established disease stage. Two dosages were analyzed, 1 × 10^6^ cells and 2.5 × 10^6^ cells. The results showed that the greatest increases in survival occurred with the administration at the pre-symptomatic stage using the lowest concentration of cells (P = 0.0015) and at a dose of 2.5 × 10^6^ for symptomatic animals (P = 0.0022).

Three studies examined the transplantation of stem cells from human fetuses into the anterior horn of the lumbar cord in immunosuppressed mice, before the onset of ALS symptoms [14, 22, 23]. In a study by Hefferan et al [22], improvement was transient and not very significant (P = 0.122), while a study by Xu et al [14] showed that a cervical and lumbar engraftment of cervical and thoracic spinal cord cells of an 8-week human fetus significantly prolonged survival by 17 days. The study was not analyzed in conjunction with the others because there were missing data.

In 2012, Lee et al [24] replaced microglia with a mutant SOD1 gene with microglia that expressed the wild-type gene using an injection of clodronate liposome and bone marrow transplants into the fourth cerebral ventricle and subsequently observed a 51.03% increase in survival.

In 2014, Sun et al reported the behavioral improvement and extended lifespan of ALS model mice transplanted with fetal human neural cells into the spinal cord. It was suggested that intrathecal transplantation of motor neurons into the lumbar spinal cord of animals migrated into the ventral horn area and improved ambulatory function and survival [25].

Also in 2014, Nizzardo et al [26] showed that survival in an animal model significantly benefited from both intrathecal and intravenous injections of specific neural stem cells that were derived from induced pluripotent stem cells. These positive effects are attributed to the activity of multiple mechanisms, including the production of neurotrophic factors and the reduction of microgliosis and macrogliosis.

In the studies described above, histopathological analyses of the nervous tissue of the spinal cord and brain were conducted after euthanasia of the animals, and a longer survival of motor neurons and glial cells was observed. Glial cells became branched after treatment but had a lower density and lower reactivity and showed less gliosis [18, 19, 21].

### Clinical studies

Table 1 [27-38] summarizes the cell therapy clinical trials. These studies were conducted with the main objective of assessing the adverse effects inherent to the procedure and the post-procedure clinical evolution of the disease. Some of these effects were assessed using the ALSFRS-R. The mean disease duration was 2.32 ± 1.1 years and ranged from 0.7 to 4.4 years. The follow-up period ranged from 6 to 47.2 months. The number of patients per study ranged from 6 to 24 with an average of 11. All but one study were uncontrolled, with a quality of C or D.

**Table 1:** Studies in Amyotrophic Lateral Sclerosis Patients With Stem Cell Therapy, 2009 - 2015

Authors	Year	N	Disease (years)	Follow-up (month)	Outcome	Survival	ALSFRS-R	Adverse events	GRADE
Glass et al [33]	2012	12	4.4	1.5	ALSFRS-R; adverse events	Not related	Stable	12	2D
Karussis et al [31]	2010	19	2.9	25	Adverse events	Not related	Fall light	Fever: 11	2D
Blanquer et al [37]	2012	11	1.8	6	Adverse events/motoneurons	Not related	Stable	Pain and paresthesia: 11	2D
Prabhakar et al [38]	2012	10	1.5	24	Survival	Not related	Decline after 3 months	No	2D
Gamez et al [30]	2010	12	2.24	47.2	Survival/tracheo/gastric tube	48 months	Declining equal to control	Fever, impaired consciousness: 1	2D
Martinez et al [27]	2009	10	2	19	Survival/tracheo/gastric tube	66 months	Improvement in 1 - 2 months and at 6 months	No	2C
Riley et al [34]	2014	6	3.7	6	Adverse events	Not related	Fall	Hemorrhage: 2	2D
Mazzini et al [28]	2012	10	0.7	24	Adverse events/mune	Not related	Fall	Pain: 7 Loss sensitive: 6	2D
Deda et al [29]	2009	13	2.6	12	Adverse events	Not related	Not ascertained	No	2D
Tarella et al [32]	2010	24	1	12	Adverse events	Not related	Not ascertained	Prolactinoma: 1 TVPO: 1	2C
Riley et al [35]	2012	12	3.1	18	Adverse events	Not related	Not ascertained	Hemorrhage: 2 reoperated	2C
Mazzini et al [28]	2012	19	1.9	108	Adverse events/mortality	52.5 months	Six patients for 74 months stabilized	No	2D
Sharma et al [36]	2015	37	Not related	60	Survival	Improved 87.76 (10.45) × 57.38 (5.31)	Fall	Minor side effects	2D

In 2009, Martinez et al [27] performed a stereotactic autologous transplantation of CD133+ mononuclear cells into the frontal cortex, noting that the procedure is safe and well tolerated in patients with ALS. The survival time of the treated patients was statistically higher (P = 0.01) than that of the control group. In that same year, Mazzini et al [28] and Deda et al [29] injected autologous bone marrow cells into the anterior horn of the thoracic and cervical spinal cord, reporting pain and sensory loss as adverse effects; there was no significant functional decline compared to the control group, which confirmed the safety of the procedure.

An observational study conducted in Spain in 2010 [30] in which the use of cell therapy in 12 patients was evaluated found no beneficial effect. In that same year, two authors [31, 32] reported the effects of simultaneous intravenous and intrathecal space injections of autologous bone marrow cells [31]. One of them used granulocyte colony-stimulating factor (G-CSF) before the procedure [32] in a study of 24 patients. Although the study addressed issues of feasibility and safety, there were signs of clinical improvement that were associated with cell mobilization following the use of G-CSF. The authors conclude that prolonged monitoring and a greater number of patients are necessary.

Recently, three separate studies [33-35] showed that the use of cells derived from fetal spinal cord or umbilical cord for microinjection into the lumbar spine after laminectomy was associated with immunosuppression. There was hemorrhage in four patients without an associated functional deterioration; the adverse effects were attributed to immunosuppression. Mazzini et al used autologous mononuclear bone marrow cell transplantation with a 9-year follow-up and observed stabilization of FVC and ALSFRS-R scores [28]. However, the percentage of young individuals in that study who had a better prognosis (above 60%) has been reported as a confounding variable.

In a retrospective analytical study, Sharma et al [36] reported a better survival with the use of mononuclear stem cells; however, there were confounding factors in the experimental group, where the improvement of survival was also related to an age below 50 years and use of lithium. In this study, there were no important adverse events.

The remaining studies [30, 37, 38] also reported good tolerance to microinjections of fetal or autologous mesenchymal cells. In conclusion, only two studies [28, 36] showed improvement of survival, but with an unsatisfactory quality.

## Discussion

Stem cell therapy is a promising potential treatment option for ALS, given the remarkable plasticity of stem cells and their ability to differentiate into multiple neuronal lineages. Stem cells can be used as important models for molecular pathway studies, drug screening, and cell therapy studies. Notably, there are two actual clinical trials, NeuralStem [39] and BrainStorm (NCT01051882).

This systematic review uses a meta-analysis to show the efficacy of stem cell therapy in improving survival in preclinical trials, whereas the number of clinical studies is still insufficient to assess their effectiveness and demonstrates only the absence of serious adverse events. However, caution is required because most clinical studies are heterogeneous with an unsatisfactory quality. Only one study was controlled but was retrospective and with confounding factors, such as the concurrent use of lithium and the interference of age in the analysis of survival.

To replace the cells that have undergone degeneration, various sources of stem cells can be used, such as bone marrow cells, neural stem cells, mesenchymal cells, astrocyte precursor cells and pluripotent cells [40]. Currently, there are basically two stem cell types for disease modeling, embryonic stem cells (ESCs) and induced pluripotent stem cells (iPSCs) [41].

The assessed preclinical trials tend to use relatively young mutant SOD1^G93A^ mice in homogeneous groups in a controlled environment in which the animals showed a similar clinical condition. Although animal models are very useful for mimicking human diseases, they have limitations because they present with a distinct disease progression and show diverse responses in trials with drugs [3, 42]. Moreover, the sample size and sex of the animals often vary between studies.

According to some authors [6, 42], there are concerns about translating preclinical studies into effective human treatments. In preclinical studies, SOD1 animal models represented familial ALS more than sporadic ALS. In addition, ALS can be defined as a syndrome in which the pathophysiological mechanisms are poorly understood [4], and it is possible that familial and sporadic ALS differs in some fundamental mechanisms that determine the effectiveness of treatments.

Recent studies with ESCs have shown that the use of cell therapy to substitute motor neurons is not sufficient to impede the neurodegenerative process. In addition to the neuronal mechanisms, the toxic environment provided by glial cells contributes to motor neuron death. On the other hand, these findings have been limited to only one gene, SOD1, but the disease involves multiple pathways involving other genes such as C90ORF72, TDP-43, FUS and cytoplasmic aggregates, suggesting an underlying convergence of cellular processes [43, 44].

ESCs are found in the blastocyst and can differentiate into oligodendrocytes, astrocytes or neurons. Hematopoietic stem cells from the bone marrow (HSCs or MSCs) produce mesenchymal cells, as well as blood cells, which are also found in adipose tissue, umbilical cord, placenta and fetal tissues. According to Vercelli et al [45], mesenchymal cells can migrate to the spinal cord of mice, where they have neuroprotective actions, such as preventing the activation of microglia and the process of tissue gliosis and improving the count of motor neurons, which explains the positive results observed in all of the animal studies and the trend observed in human studies.

Another option for transplantation could be the use of stem cells that are derived from the olfactory epithelium (OECs). OECs continue to multiply during the postnatal period, are multipotent, and serve as conductive connections between the central and peripheral nervous systems [46]. In 2008, a clinical trial of 35 patients was conducted [47] and found that olfactory cell transplantation may slow disease progression. OEC transplantation for ALS has been performed in China with positive effects in spinal cord injury studies, such as axonal regeneration, remyelination and functional improvements. Although a large Chinese study reports that OECs may offer a benefit to ALS, other reports criticize the observed outcomes and do not support the clinical translation of this therapeutic approach at this time.

The histological analyses performed in all of these cell therapy studies show an improved animal survival, which supports the potential of this approach for neuroprotection with greater tissue preservation. However, among both cell therapy preclinical trials and cell therapy clinical trials, there were no studies with negative results, which may indicate a possible publication bias.

The development of iPSCs has led to remarkable changes in stem cell science. This technology has made it possible to obtain pluripotent stem cells directly from a patient’s adult cells. These cells are usually induced to form embryonic bodies and subsequently form neural precursor cells (NPCs) [41], which holds new promise for the treatment of neurodegenerative diseases.

However, studies have shown that there are many similarities between iPSCs and ESCs, such as telomere renewal during cell reprogramming into iPSCs and telomere shortening upon differentiation into somatic cells [48]. This similarity suggests that iPSCs could potentially be used as patient-specific ESCs, consequently preventing rejection and eliminating any ethical issues.

These recent studies in humans with ALS are summarized in Table 1 and include many differences, such as the number of patients, cell type, delivery method and outcome measurement strategies; however, each study has the potential to contribute to increasing our current understanding of the safety and feasibility of stem cell therapies for ALS. These studies were considered to be of low quality because of biases.

Moreover, in most studies, the cell therapy procedure was uncontrolled and performed in patients with a very advanced stage of disease. The disease onset was variable and frequently prolonged at 2.32 ± 1.1 years. Most authors agree that the treatment must be performed early in the course of the disease [41]. The goal of most of the studies was to assess adverse events and tolerability to treatment.

Guidelines have been introduced [49] that should reduce the number of false positives in preclinical studies and therefore prevent unnecessary clinical trials, which have occurred for various drugs. These recommendations include the following: 1) rigorously assessing an animal’s physical and biochemical characteristics with respect to human disease; 2) characterizing disease symptoms and the occurrence of death and being alert to unexpected variations; and 3) creating a mathematical model to address questions about the experimental design, such as the number mice that must be included in a study. To reduce concerns about animal research, Perrin [42] suggested excluding irrelevant animals, balancing for gender, avoiding the use siblings in the same treatment group, and tracking genes that induce non-inherited disease.

In conclusion, ALS is a rare heterogeneous disease that is still poorly understood in terms of its pathophysiology. Moreover, from a clinical point of view, ALS is difficult to manage. Preclinical studies of stem cell therapy show great efficacy, whereas more prospective and controlled studies are needed to establish the effectiveness of clinical studies in improving survival. Nonetheless, the most effective cell type to be used in transplantation must be determined, and it should be the one that shows better potential for neurogenesis and not only neuroprotective mechanisms.
